# Anti-biofilm Activities from Resveratrol against *Fusobacterium nucleatum*

**DOI:** 10.3389/fmicb.2016.01065

**Published:** 2016-07-05

**Authors:** Zhiyan He, Zhengwei Huang, Wei Zhou, Zisheng Tang, Rui Ma, Jingping Liang

**Affiliations:** Department of Endodontics, Shanghai Research Institute of Stomatology, Ninth People’s Hospital, Shanghai Jiao Tong University School of Medicine, Shanghai Key Laboratory of StomatologyShanghai, China

**Keywords:** *F. nucleatum*, resveratrol, biofilm, gene expression, antimicrobials

## Abstract

*Fusobacterium nucleatum* is a Gram-negative, anaerobic bacterium that plays an important role in dental plaque biofilm formation. In this study, we evaluate the effect of resveratrol, a phytoalexin compound, on *F. nucleatum* biofilm formation. The effects of different concentrations of resveratrol on biofilms formed on 96-well microtiter plates at different time points were determined by the MTT assay. The structures and thicknesses of the biofilm were observed by confocal laser scanning microscopy (CLSM), and gene expression was investigated by real-time PCR. The results showed that resveratrol at sub-MIC levels can significantly decrease biofilm formation, whereas it does not affect the bacterial growth rate. It was observed by CLSM images that the biofilm was visually decreased with increasing concentrations of resveratrol. Gene expression was down regulated in the biofilm in the presence of resveratrol. Our results revealed that resveratrol can effectively inhibit biofilm formation.

## Introduction

In natural and industrial environments, bacteria mostly grow as biofilms attached to surfaces or are associated with interfaces, where bacterial cells are encased in a self-produced extracellular polymeric matrix. Biofilm formation is a complex and multifactorial process that involves at least two steps. The first step involves the adherence of the bacterial cells to the host surface; subsequently, adherent cells form a multilayer biofilm covered by an extracellular matrix (Flemming and Wingender, 2010; Trappetti et al., 2011; Srinandan et al., 2015). The microorganisms in the biofilm undergo profound changes during the shift from a planktonic lifestyle. After incorporation into the biofilm, microorganisms are resistant not only to host defense mechanisms, such as phagocytosis, but also to antimicrobial agents. This change has been suggested to be involved in the persistence of infection by such microorganisms (Costerton et al., 1999; Jacqueline and Caillon, 2014).

Dental plaque biofilms are composed of a very complex mixture of species of bacteria, and their formation is highly ordered, starting with the initial colonizing bacteria attaching to the tooth surface with the acquired pellicle and subsequent coaggregation of late colonizers. More than 700 bacterial species have been detected in the human oral cavity. Among them, *Fusobacterium nucleatum* is frequently isolated from both supra- and sub-gingival dental plaque biofilms in humans. *F. nucleatum* is a Gram-negative, anaerobic, non-motile, non-spore-forming, spindle-shaped, or fusiform rod bacterium. It plays an important role in the development of complex dental plaque biofilms as a bridge bacterium interaction with early and late colonizing bacteria in the oral cavity (Kolenbrander et al., 2006; Sasaki-Imamura et al., 2010; Tian et al., 2010; Ali Mohammed et al., 2013).

The worldwide practice of indiscriminate and continuous use of antibiotics for the control and prophylaxis of bacterial pathogens has led to the development of bacterial resistance to most available antimicrobials. Antibiotics used as antibiotic growth promoters by adding continuously to animal feeds in very low amounts also play a significant role in the emergence of resistant bacteria. The rising number of infections caused by bacterial isolates resistant to conventional antibiotics has led to an intense search for novel antimicrobials and chemotherapeutics, including natural chemicals and natural plant products, which have generated increased interest with regard to their potential for use in treating infectious diseases (van den Bogaard and Stobberingh, 2000; Molhoek et al., 2011; de Lima Pimenta et al., 2013). Resveratrol (*trans*- 3,5,4′-trihydroxystilbene) is a naturally derived polyphenol natural product that is mainly found in the skin of grapes, berries, and so on and is also a component of red wine. Resveratrol has a broad range of biological effects, which include antimicrobial, antioxidant, antiinflammatory, antiaging, anticarcinogenic, and neuroprotective effects (Augustine et al., 2014; Lee K. et al., 2014). As a natural product, resveratrol may be an effective biofilm inhibitor. Recent studies have indicated that resveratrol inhibits the biofilm formation of some bacterial pathogens, including *Vibrio cholerae, Staphylococcus aureus, Escherichia coli O157:H7*, and *Pseudomonas aeruginosa* (Augustine et al., 2014; Lee J.H. et al., 2014; Qin et al., 2014).

There has been increasing interest in natural products as agents for preventing oral diseases, particularly dental biofilm-related diseases (Sintim and Gursoy, 2016). Millhouse et al. (2014) have already studied the effect of resveratrol on multi-species biofilm containing *Streptococcus mitis, F. nucleatum, Porphyromonas gingivalis*, and *Aggregatibacter actinomycetemcomitans*. Their results showed that resveratrol did not appear to have bactericidal properties against multi-species biofilms (Millhouse et al., 2014).

Therefore, in this study, we investigated the effect of resveratrol on biofilm formation by *F. nucleatum*. We mainly focused on the effects of resveratrol on the aggregation of bacteria, different stages of biofilm formation, biofilm structure and gene expression. To our knowledge, this is the first report describing the effect of resveratrol on *F. nucleatum* aggregation, biofilm formation and structure. This research would offer the possibility of the use of natural product resveratrol to prevent *F. nucleatum* biofilm infections. It may useful in the development of natural product as novel antimicrobial agents to treat and prevent of dental diseases.

## Materials and Methods

### Bacterial Strains and Growth Conditions

The *F. nucleatum* ATCC10953 strain was provided by the State Key Laboratory of Oral diseases, West China Hospital of Stomatology, Sichuan University. Planktonic and biofilm forms of the *F. nucleatum* ATCC10953 strain were grown in Brain Heart Infusion Broth (BHI; Difco Laboratories, Sparks, MD, USA) at 37°C under anaerobic conditions (80% N_2_, 10% CO_2_, and 10% H_2_).

### Effects of Resveratrol on Planktonic Cell Growth

The *F. nucleatum* ATCC10953 strain was used to anaerobically inoculate a fresh BHI culture with different concentrations of resveratrol (0, 12.5, 25, 50, 100 μg ml^-1^) at 37°C without agitation. The optical density at 600 nm was measured using a spectrophotometer (UV1601, Shimadzu, Japan) at different time intervals. The experiment was replicated three times with triplicate samples at each time point.

### Effects of Resveratrol on Biofilm Formation

To quantify *F. nucleatum* biofilm growth, we applied the MTT [3-(4,5-dimethylthiazol-2-yl)-2,5-diphenyl tetrazolium bromide] assay in 96-well polystyrene plates (He et al., 2012). An overnight culture at a final concentration of 10^6^ CFU/ml was added to 200 μl of fresh BHI liquid medium in each flat-bottom well with different concentrations of resveratrol (0, 1.5625, 3.125, 6.25, 12.5, 25 μg ml^-1^). The plates were then incubated at 37°C for different times (24, 36, 48 h) without agitation. The culture was then removed, and the wells carefully washed three times with sterile phosphate-buffered saline (PBS) to remove non-adherent cells. The cultures were stained with 100 μl of MTT (5 mg ml^-1^) for 3 h in a dark place and washed three times with PBS. Next, 100 μl of lysing solution [10% (v/v) sodium dodecyl sulfate and 50% (v/v) dimethylformamide in distilled water] was added to dissolve the biofilm for 3 h at room temperature before reading the OD_590__nm_ values. All of the experiments were performed in triplicate with at least three replicates, and wells without cells were used as blank controls.

### Aggregation Assays

Aggregation experiments were performed as previously described with minor modifications (He et al., 2015). Briefly, *F. nucleatum* was grown overnight in BHI broth with different concentration of resveratrol (0, 1.5625, 3.125, 6.25, 12.5, 25 μg ml^-1^). The bacteria were harvested by centrifugation at 13,400 *g* for 30 s, washed twice with PBS, and resuspended in PBS to an optical density of approximately 0.6 at 600 nm, as determined by using a spectrophotometer. All of the samples were incubated at 37°C for 150 min, and the OD_600__nm_ was recorded at different time intervals (0, 20, 40, 60, 90, 120, and 150 min). Before measurement, the samples were equilibrated at room temperature for 5 min. The percentage of aggregation was calculated as follows: (OD_600__nm_ at time zero – OD_600__nm_ at time x min)/(OD_600__nm_ at time zero) × 100%. All of the experiments were performed in triplicate.

### Effects of Resveratrol on the Biofilm Structure

*Fusobacterium nucleatum* biofilms were formed on glass-bottom chamber slides with different concentrations of resveratrol (0, 1.5625, 3.125, 6.25, 12.5, 25 μg ml^-1^) for 24 h at 37°C. The biofilms formed on each sheet were washed twice with saline to remove unbound cells and stained for 30 min in the dark with L-7012 LIVE/DEAD BacLight TM bacterial cells containing SYTO 9 dye and propidium iodide (Molecular Probes, Inc., Eugene, OR, USA). A confocal laser scanning microscope (CLSM; Leica TCS SP2, Leica microsystems, Germany) was used to record confocal image stacks in five random locations near the center of each slide. Five confocal data sets were recorded at 40× magnification with a numerical aperture of 1.25 and Leica confocal software was analyzed for the thicknesses of the biofilms and bacterial vitality, and the average and standard deviation were calculated. In each experiment, the exciting laser intensity, background level, contrast, and electronic zoom were maintained at the same level.

### RNA Extraction, Reverse Transcription, and qPCR

The biofilms that formed with different concentrations of resveratrol (0, 12.5, 25 μg ml^-1^) at 24 h were harvested by centrifugation, resuspended in Trizol reagent (Takara) and transferred to an RNase-free 1.5 ml microcentrifuge tube. Total RNA extractions were performed according to the manufacturer’s instructions. Purified RNA was dissolved in 20 μl of DEPC-treated water and stored at -80°C until required for cDNA labeling. A cDNA synthesis kit (Takara) was used to generate cDNA. The reverse transcription reaction mixture (20 μl) containing 4 μl of 5× buffer (containing dNTP and Mg^2+^), 1 μl of PrimeScript RT Enzyme Mix I, 1 μl of Oligo dT primer, 1 μl of random hexamers, and 1 μg of an RNA sample, was incubated at 37°C for 15 min and the reaction was terminated at 85°C for 5 s according to the manufacturer’s instructions. The cDNA samples were stored at -20°C until use.

The qPCR reaction mixture (20 μl) contained 1× SYBR Green PCR master mix, 5 μl of template cDNA, and 0.5 μM of the appropriate forward and reverse PCR primers. qPCR conditions included an initial denaturation at 98°C for 5 min, followed by a 40-cycle amplification consisting of denaturation at 98°C for 15 s, annealing at 50°C for 15 s, and extension at 72°C for 30 s. The resulting cDNA and negative control were amplified using an Applied Biosystems 7900HT Fast Real Time PCR System (Applied Biosystems). The expression levels of all of the tested genes (**Table 1**) as determined by qPCR were normalized using the 16S rRNA gene of *F. nucleatum* as an internal standard (Lee et al., 2011). Each assay was performed with three independent RNA samples in triplicate. The fold changes of the expression levels were using the ΔΔ*C_q_* method.

**Table 1:** Nucleotide sequences of primers used in this study.

Primer	Primer sequence (5′–3′)	Target
Fn 16S F	AAGCGCGTCTAGGTGGTTATGT	16S rRNA
Fn 16S R	TGTAGTTCCGCTTACCTCTCCAG	16S rRNA
Fn 0116 RT-F	GTATCCCTGCTGCTCCAA	FN0116
Fn 0116 RT-R	GTGCTTCTGCTTCCTTAGTC	FN0116
Fn 0132 RT-F	CCAATGCCACTGATGAACCT	FN0132
Fn 0132 RT-R	CAGCAGCTGAGACAGCATTG	FN0132
Fn 0503 RT-F	TCACCCTTGAGATTTCCTTT	FN0503
Fn 0503 RT-R	GAAGTTGCAAAGGCTAAAAGC	FN0503
Fn 0659 RT-F	GTTGGAGCAACACCAGTTCC	FN0659
Fn 0659 RT-R	CCAAGTGGTTCAACATGCAC	FN0659
Fn 0675 RT-F	ATTGACCCAGCAAAAGTTAC	FN0675
Fn 0675 RT-R	GGCATCATTCCACCAGCA	FN0675
Fn 1856 RT-F	TCTGCTGCTGTTGTTGCTTT	FN1856
Fn 1856 RT-R	GGGTGGAGCAATGGACTTAG	FN1856

### Statistical Analysis

Analysis of variance (ANOVA) with *post hoc* test was used to calculate the significance of the difference between the biofilms formed by *F. nucleatum* with or without resveratrol under the tested conditions (SPSS 15.0 software, USA). *P* < 0.05 was considered statistically significant.

## Results

### Effects of Resveratrol on Planktonic Cell Growth

**FIGURE 1:**
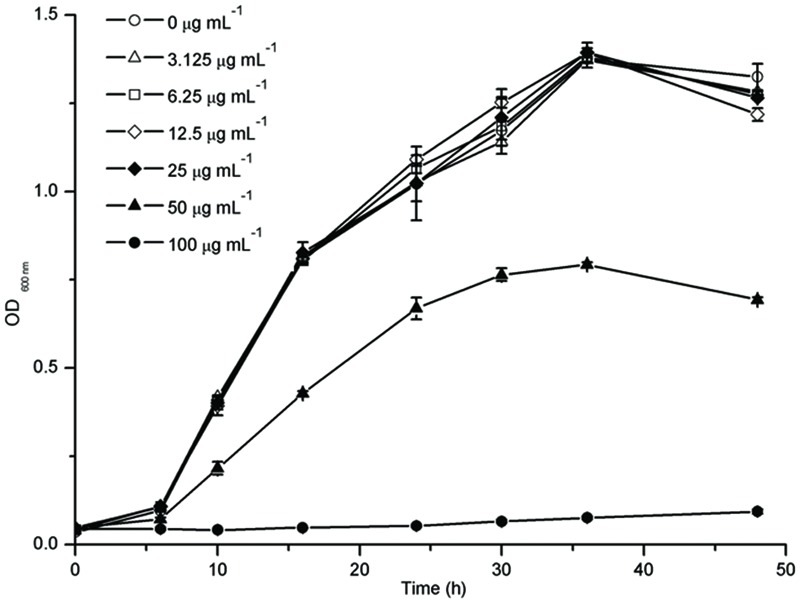
**Planktonic Cell Growth of *Fusobacterium nucleatum* with different concentrations of resveratrol**.

The growth of *F. nucleatum* with different concentrations of resveratrol over a time course showed the bacteriostatic effect of resveratrol. It was observed that in comparison to the control, the bacterial growth was significantly inhibited with 50 μg ml^-1^ resveratrol treatment (**Figure 1**). The MIC for *F. nucleatum* using resveratrol was recorded as 100 μg ml^-1^. However, there was no obvious difference in the growth curve with resveratrol concentrations below 25 μg ml^-1^.

### Effects of Resveratrol on the Biofilms

**FIGURE 2:**
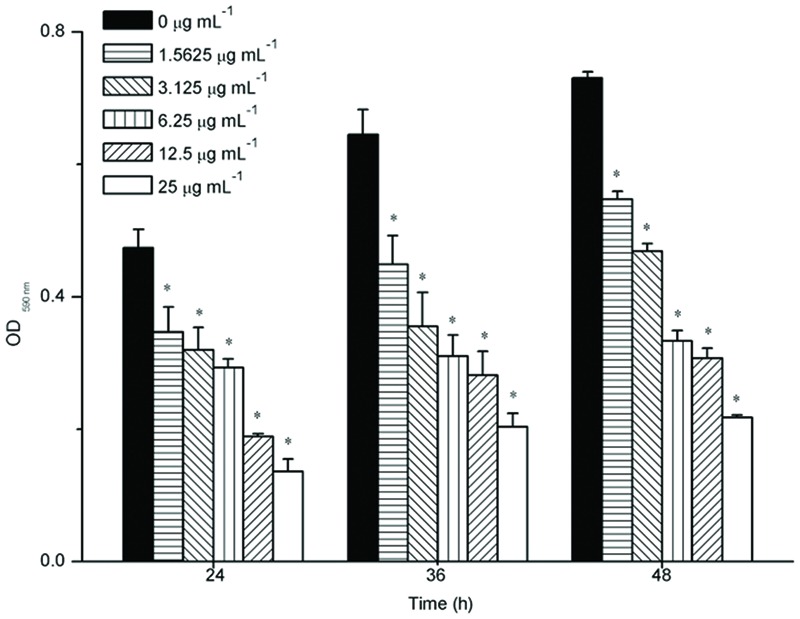
**Biofilm formation of *F. nucleatum* with different concentrations of resveratrol at different time points.** Statistically significant differences (^∗^*P* < 0.05) between with or without resveratrol at different time points.

The effect of resveratrol on biofilm formation was evaluated when *F. nucleatum* was grown in 96-well plates of polystyrene. Sub-MIC levels of resveratrol (0–25 μg ml^-1^) were supplemented in the media from the onset of incubation with *F. nucleatum* (**Figure 2**). After 24 h, *F. nucleatum* exhibited OD _590nm_ values of 0.474 ± 0.028. With increasing concentrations of resveratrol, the biofilms formed by *F. nucleatum* exhibited OD_590nm_ values that decreased from 0.347 ± 0.038 at 1.5625 μg ml^-1^ to 0.136 ± 0.018 at 25 μg ml^-1^ after a 24 h incubation. Similar trends were observed after 36 and 48 h. The biofilms formed by *F. nucleatum* exhibited OD _590nm_ values of 0.645 ± 0.038 and 0.730 ± 0.010 at 36 and 48 h, respectively, whereas in the presence of resveratrol, the values decreased from 0.449 ± 0.043 at 1.5625 μg ml^-1^ to 0.203 ± 0.020 at 25 μg ml^-1^ after a 36 h incubation and decreased from 0.548 ± 0.012 at 1.5625 μg ml^-1^ to 0.217 ± 0.005 at 25 μg^-^ml^-1^ after a 48 h incubation, respectively. It was shown that resveratrol could effectively inhibit biofilm formation at different time points.

### Effects of Resveratrol on Aggregation

**FIGURE 3:**
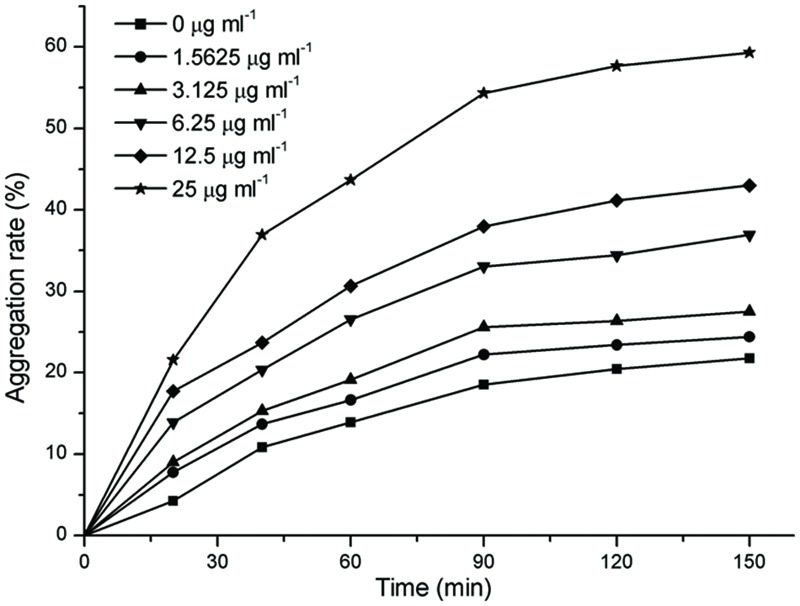
**The extents of aggregation of *F. nucleatum* with different concentrations of resveratrol.** Representative data are shown, and the experiment was repeated three times with similar results.

The aggregation of *F. nucleatum* is shown in **Figure 3**. In general, the extents of aggregation increased with the increasing time, but the extents of aggregation mildly increased after 90 min. As shown in **Figure 3**, the extents of aggregation of *F. nucleatum* without resveratrol reached 21.75% after 150 min. The extents of aggregation with 1.5625 and 3.125 μg ml^-1^ resveratrol was slightly increased compared to bacteria grown without resveratrol. However, the extents of aggregation noticeably increased when the concentration of resveratrol reached 6.25 μg ml^-1^. *F. nucleatum* quickly aggregated with 25 μg ml^-1^ resveratrol, and cell aggregation was apparent by visual inspection. The maximum extents of aggregation for *F. nucleatum* reached 59.31% with 25 μg ml^-1^ resveratrol after 150 min.

### Effects of Resveratrol on the Biofilm Structure

**FIGURE 4:**
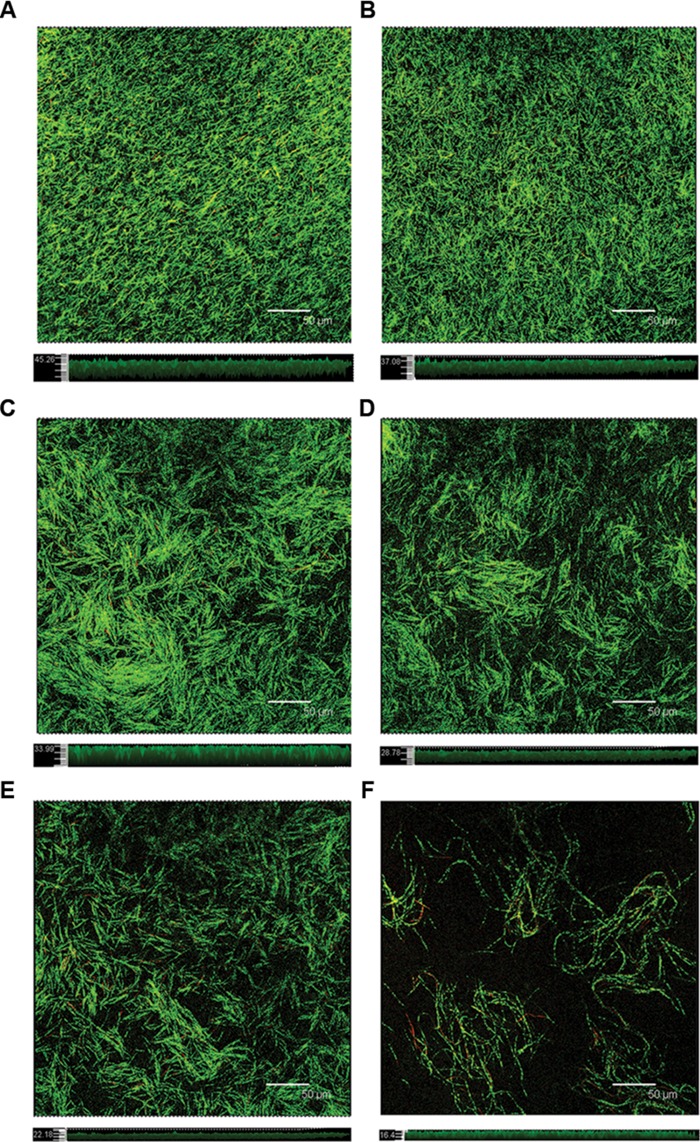
**Confocal laser scanning micrographs of the biofilm formed with different concentrations of resveratrol.** Top sections represent the X–Y panels, and bottom sections represent the Z-scales, respectively. **(A)** 0 μg ml^-1^; **(B)** 1.5625 μg ml^-1^; **(C)** 3.125 μg ml^-1^; **(D)** 6.25 μg ml^-1^; **(E)** 12.5 μg ml^-1^; **(F)** 25 μg ml^-1^. Bar = 50 μm.

The surface area of glass-bottom chamber slides that were covered with a *F. nucleatum* biofilm was characterized using CLSM after 24 h of incubation (**Figure 4**). In the absence of resveratrol, the biofilm formed by *F. nucleatum* had a uniform distribution with complete coverage of the attached surface. The biofilm appeared loose and tended to gather into relatively small aggregates that were easily discernible in accordance with resveratrol added to the culture. The biofilm thickness after 24 h of cultivation without resveratrol was observed to be up to 46.171 ± 3.369 μm. The biofilm thickness became thinner with increasing concentrations of resveratrol, and it was only approximately 16.811 ± 1.626 μm at a 25 μg ml^-1^ concentration of resveratrol (*P* < 0.05). We also calculated that the proportion of viable (green) cells among all cells was 84.15 ± 1.69%, 82.19 ± 3.88%, 84.53 ± 1.56%, 85.48 ± 1.77%, 85.25 ± 2.47%, 84.71 ± 2.85% with different resveratrol treatment (0, 1.5625, 3.125, 6.25, 12.5, 25 μg ml^-1^), respectively.

### Gene Expression in the Biofilms

**FIGURE 5:**
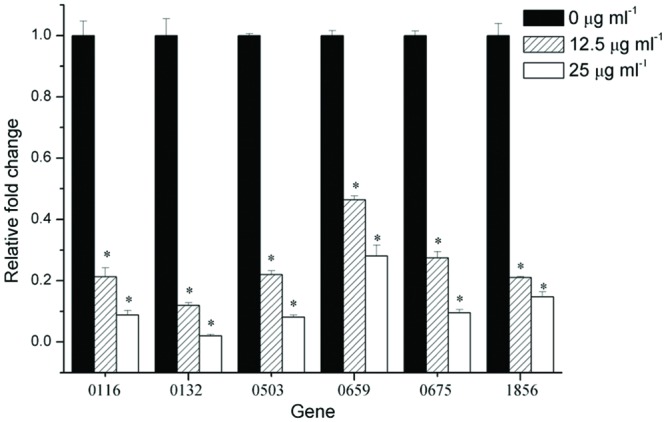
**Gene expression of *F. nucleatum* strain biofilms.** The results represent the means and SDs of at least three independent experiments performed in triplicate. Statistically significant differences (^∗^*P* < 0.05) in gene expression between different concentrations.

To gain insight into gene expression, real-time PCR analysis was used to quantify the effect of 12.5 and 25 μg ml^-1^ resveratrol on the biofilms formed by *F. nucleatum*. The selected genes including FN0116 and FN0675 encoding stress-induced proteins, FN0132 encoding hemolysin, FN0503 encoding LysR-family transcriptional regulator, FN0659 encoding the ABC transporter substrate binding protein and FN1856 encoding butyrate-acetoacetate CoA-transferase subunit were considered to be important virurence factors (Skar et al., 2003; Lee et al., 2011). In general, six tested genes (**Table 1**) were down-regulated in the biofilms grown with resveratrol compared to cells grown without resveratrol (**Figure 5**). Additionally, the relative fold changes of the levels of the gene transcripts decreased with increasing resveratrol concentration. Transcription of FN0132 in the biofilms formed by *F. nucleatum* were significantly downregulated after 12.5 and 25 μg ml^-1^ resveratrol treatment, with the relative change being 0.12- and 0.02-fold, respectively. The expression of other down-regulated genes in the biofilms with resveratrol, including FN0116, FN0503, FN0659, FN0675 and FN1856, were reduced, ranging from approximately 0.46- to 0.21-fold at 12.5 μg ml^-1^ and from approximately 0.28- to 0.08-fold at 25 μg ml^-1^. We also compared the gene expression of planktonic cultures and the result showed that there was no obvious difference in gene expression between planktonic cultures by *F. nucleatum* with or without resveratrol (*P* > 0.05) (**Supplementary Figure S1**).

## Discussion

*Fusobacterium nucleatum* is commonly cultivated from the subgingival plaque of periodontitis patients, and because of its ability to congregate with many oral bacteria, it functions as a bridge between early and late colonizers in the dental plaque biofilm (Ali Mohammed et al., 2013). Biofilm formation regulates *F. nucleatum* survival and invasiveness including surviving in an aerobic environment, invading the epithelium. *F. nucleatum* also decrease in membrane permeability to resist antimicrobial stress (Gursoy et al., 2010; Keskin et al., 2014).

At the start of this study, we investigated the effect of resveratrol on bacterial growth. Our results demonstrated that resveratrol did not significantly affect the growth rate of the bacteria at concentrations up to 50 μg ml^-1^. Thus, we studied whether resveratrol can affect biofilm formation at sub-MIC concentrations through MTT assays. This assay is to convert the yellow tetrazolium salt MTT to intracellular insoluble purple formazan by metabolically active cells. The MTT assay has been used to detect the effect of antibiofilm agents in the biofilm formation in many previous studies (Chusri et al., 2012; He et al., 2012; de Lima Pimenta et al., 2013). Therefore, we used this assay for the quantification of viable biofilm bacteria and the result was shown that resveratrol attenuated the biofilm formation more effectively when the concentration increased, ranging from 1.5625 to 25 μg ml^-1^. No significant planktonic cell growth rate inhibition was detected at concentrations that inhibited biofilm formation. Our results suggested that the mechanisms by which resveratrol exerts its effects on *F. nucleatum* biofilms at sub-MICs were distinctly different from the mode of action of resveratrol for growth inhibition. According to the CLSM images, the thickness of biofilms after 24 h of cultivation was thinner with increasing concentrations of resveratrol. We also found that the proportion of viable (green) cells among all cells following resveratrol treatment was similar to the proportion without treatment, as determined through CLSM by staining with SYTO9/PI (a DNA-binding dye that stains both living and dead cells). Therefore, unlike antibiotics that are used to inhibit cell growth, resveratrol may be identified as a biofilm inhibitor that does not affect bacterial growth.

The ability of bacterial cells to come into contact and form aggregates, or autoaggregation is considered important for colonization of host cells in pathogenic bacteria (Lee et al., 2010; Abdel-Nour et al., 2014). During biofilm development, autoaggregation is a process through which a strain within the biofilm produces polymers to boost the integration of genetically identical strains, and is also a prerequisite of biofilm formation (Zhang et al., 2004; Das et al., 2010; Nyenje et al., 2012). However, the results of the present study showed that resveratrol increased *F. nucleatum* aggregation but decreased biofilm formation which were consistent with previous study concerning the effects of proanthoyanides on *Staphylococcus epidermidis* biofilm formation. Trentin et al. (2015) thought that the proposed mechanism of bacterial attachment inhibition was based on electrostatic repulsion and changes in hydrophilicity. This result is also in agreement with our observation from CLSM fluorescence imaging, which revealed that the biofilm changed from confluent and more evenly distributed to gather into distinct, easily discernible clusters after adding resveratrol.

Our data show that six selected genes were down-regulated after adding resveratrol in the biofilm formation. Among them, the ABC transporter substrate binding protein, which is encoded by FN0659, is a ubiquitous integral membrane protein that translocates from a variety of substrates, ranging from ions to macromolecules, either out of or into the cytosol (hence, they are defined as importers or exporters, respectively; Yu et al., 2015). It is also responsible for autoinducer 2 uptake into the cell which may play an important role in the *F. nucleatum* quorum sensing (QS) system (Rezzonico and Duffy, 2008). In previous research, a novel ABC transporter was identified in *R. leguminosarum* and required for biofilm formation. Seaton et al. (2011) predicted that *SMu0836* and *SMu0837* encoded ABC transporters and constructed a mutant strain *Δ836p* in which a kanamycin resistance cassette replaced the *SMu836* gene. They also found that the *Δ836p* strain had an impaired capacity to form biofilms (Vanderlinde et al., 2010; Seaton et al., 2011). The LysR family of transcriptional regulators encoded by FN0503 represents the most common type of transcriptional regulators in bacteria. Members of this family have a conserved structure with an N-terminal DNA-binding helix–turn–helix motif and a C-terminal co-inducer-binding domain. LysR-Type transcriptional regulators were among the most common types of positive regulators in prokaryotes. They have previously been shown to affect virulence, QS and biofilm formation. An *oxyR* (a LysR-Type Regulator)-defective mutant was constructed and impaired in biofilm formation in comparison to the parental strain (Bernier et al., 2008; Maddocks and Oyston, 2008; Hennequin and Forestier, 2009). Stress-induced proteins, such as DnaK (FN0116) and GroEL (FN0675) molecular chaperones, are considered to be heat shock proteins (Hsp) and participate in a variety of cellular processes including protein folding, protein trans-location, and the assembly/disassembly of protein complexes. These stress-related genes are central for tolerance to environmental stresses and are important for QS. In previous studies, Lemos et al. (2007) created a knockdown strategy to lower the levels of DnaK by over 95% in strain SM12 and the level of GroEL about 80% in strain SM13. They found that, SM12 and SM13 had impaired biofilm-forming capacities compared with the wild-type strain (Yuan et al., 2005; Lemos et al., 2007). The key virulence factor, hemolysin (FN0132) caused lysis of erythrocytes, which provide iron and create an anaerobic environment by reducing the oxygen supply to the site of infection in Fusobacteria (Miao et al., 2010). Alizarin could inhibit *S. aureus* and *S. epidermidis* biofilm formation and repress expression of their hemolysin gene. Gowrishankar et al. (2012) found that two coral-associated bacterial (CAB) extracts reduced the production of EPS and hemolysin, which ultimately resulted in the significant inhibition of biofilms formed by both methicillin-resistant and -susceptible *S. aureus* (Lee et al., 2016). In addition, butyrate-acetoacetate CoA-transferase subunit B (FN1856) is suggested to be the most important route for the production of butyrate which is present in elevated levels in plaques associated with periodontitis, and may have the ability to penetrate the gingival epithelium (Bolstad et al., 1996; Hippe et al., 2011). These observations are consistent with our results that show that these genes were down-regulated in the presence of resveratrol. This may be the reason that biofilm formation was attenuated after adding resveratrol.

Evidence is accumulating showing that the ability to form biofilms in many organisms involves QS regulation. QS is a general cell–cell communication mechanism in the bacterial kingdom occurring via small diffusible signal molecules called autoinducers (AI), which act as members of a chemical “language” to coordinate bacterial population behaviors. Through the accumulation of bacterially produced AI, the bacterial population is able to sense increases in cell density and alter their gene expression accordingly to optimize their physiological response for a particular environmental stimulus and thereby modify the bacterial phenotype (Krysciak et al., 2011; Pei and Lamas-Samanamud, 2014). Therefore, we speculated that the natural compound resveratrol might attenuate *F. nucleatum* biofilm formation by disturbing its QS system, which was consistent with previous findings (Qin et al., 2014).

A wide variety of natural products have attracted considerable research interest because of their potential value in disease prevention and treatment. There is increasing demand from the public for natural products that can achieve desired antimicrobial and antiinflammatory effects for oral health care to complement and enhance the mechanical removal of plaque biofilms (Chen et al., 2011). Resveratrol, a natural compound found in some foods and drinks, inhibits the biofilm formation of some bacterial pathogens. Our study showed that resveratrol also inhibited *F. nucleatum* biofilm formation. However, Millhouse et al. (2014) have already found the resveratrol did not affect multi-species biofilm composition which seemed to be in disagreement with ours. The reason may be that the multi-species biofilm contains four bacteria including *S. mitis, F. nucleatum, P. gingivalis*, and *A. actinomycetemcomitans*. Among them, *S. mitis* was the most dominant species (83.24%) in mature biofilm and the percentage of *F. nucleatum* was only 15.16%. Since resveratrol can be used to treat or prevent *F. nucleatum* infection, its toxicity to mammalian cells needs to be clarified. Babich et al. (2000) found that the midpoint cytotoxicity values for a 24 h exposure to resveratrol were 400 μM (≈100 μg ml^-1^) for normal fibroblasts isolated from the oral cavity. The inhibition concentration used in our study was only 1.5625–25 μg ml^-1^. Thus, resveratrol has little effect on normal human cells at this concentration. These data support that natural product resveratrol has developed non-aggressive and non-toxic therapeutic strategies to regulate biofilm formation. Therefore, resveratrol is considered to be a potential therapeutic target by attenuating the capacity of pathogenic bacteria to cause infection and unlikely to induce antimicrobial drug resistance.

## Conclusion

Our study highlights that resveratrol has an inhibitory effect on *F. nucleatum* biofilm formation. The significant advantage of using resveratrol is that it does not affect the growth and survival of pathogenic bacteria at usable concentrations, thus avoiding selective pressure and the induction of resistance. Thus, resveratrol could be further explored as a means to prevent dental plague accumulation or *F. nucleatum* infection. However, natural biofilms as dental plaque are complex structures of multiple organisms which may behave quite differently from monoculture biofilms. Thus, further studies will contribute to understanding the molecular mechanism underlying the inhibitory effect of resveratrol on other oral bacteria biofilm formation, and possibly provide therapeutic or preventative methods for dental diseases.

## Author Contributions

JL: designed this study. ZHe: drafted paper and conducted the experiment. ZHu: revised the paper critically. WZ, ZT, and RM: do the acquisition, analysis, or interpretation of data. We declare that all listed authors have made substantial contributions. We also declare that nobody who qualifies for authorship has been excluded from the list of authors.

## Conflict of Interest Statement

The authors declare that the research was conducted in the absence of any commercial or financial relationships that could be construed as a potential conflict of interest.

## Supplementary Material

FIGURE S1 **Gene expression of *F. nucleatum* strain planktonic cultures.** The results represent the means and SDs of three independent experiments performed in triplicate. No significant differences (*P* > 0.05) in gene expression between different concentrations.

## References

[B1] Abdel-Nour M. Duncan C. Prashar A. Rao C. Ginevra C. Jarraud S. (2014). The *Legionella pneumophila* collagen-like protein mediates sedimentation, autoaggregation, and pathogen-phagocyte interactions. *Appl. Environ. Microbiol.* 80 1441–1454. 10.1128/AEM.03254-13 24334670 PMC3911070

[B2] Ali Mohammed M. M. Nerland A. H. Al-Haroni M. Bakken V. (2013). Characterization of extracellular polymeric matrix, and treatment of *Fusobacterium nucleatum* and *Porphyromonas gingivalis* biofilms with DNase I and proteinase K. *J. Oral Microbiol.* 5 10.3402/jom.v5i0.20015 PMC3559756 23372876

[B3] Augustine N. Goel A. K. Sivakumar K. C. Kumar R. A. Thomas S. (2014). Resveratrol–a potential inhibitor of biofilm formation in *Vibrio cholerae*. *Phytomedicine* 21 286–289. 10.1016/j.phymed.2013.09.010 24182988

[B4] Babich H. Reisbaum A. G. Zuckerbraun H. L. (2000). In vitro response of human gingival epithelial S-G cells to resveratrol. *Toxicol. Lett.* 114 143–153. 10.1016/S0378-4274(99)00288-X 10713479

[B5] Bernier S. P. Nguyen D. T. Sokol P. A. (2008). A LysR-type transcriptional regulator in *Burkholderia cenocepacia* influences colony morphology and virulence. *Infect. Immun.* 76 38–47. 10.1128/iai.00874-07 17967860 PMC2223663

[B6] Bolstad A. I. Jensen H. B. Bakken V. (1996). Taxonomy, biology, and periodontal aspects of *Fusobacterium nucleatum*. *Clin. Microbiol. Rev.* 9 55–71. 8665477 10.1128/cmr.9.1.55 PMC172882

[B7] Chen Y. Wong R. W. Seneviratne C. J. Hagg U. Mcgrath C. Samaranayake L. P. (2011). The antimicrobial efficacy of Fructus mume extract on orthodontic bracket: a monospecies-biofilm model study in vitro. *Arch. Oral Biol.* 56 16–21. 10.1016/j.archoralbio.2010.08.006 20864088

[B8] Chusri S. Sompetch K. Mukdee S. Jansrisewangwong S. Srichai T. Maneenoon K. (2012). Inhibition of *Staphylococcus epidermidis* biofilm formation by traditional thai herbal recipes used for wound treatment. *Evid. Based Complement. Alternat. Med.* 2012:159797 10.1155/2012/159797 PMC3420317 22919409

[B9] Costerton J. W. Stewart P. S. Greenberg E. P. (1999). Bacterial biofilms: a common cause of persistent infections. *Science* 284 1318–1322. 10.1126/science.284.5418.1318 10334980

[B10] Das T. Sharma P. K. Busscher H. J. Van Der Mei H. C. Krom B. P. (2010). Role of extracellular DNA in initial bacterial adhesion and surface aggregation. *Appl. Environ. Microbiol.* 76 3405–3408. 10.1128/AEM.03119-09 20363802 PMC2869138

[B11] de Lima Pimenta A. Chiaradia-Delatorre L. D. Mascarello A. De Oliveira K. A. Leal P. C. Yunes R. A. (2013). Synthetic organic compounds with potential for bacterial biofilm inhibition, a path for the identification of compounds interfering with quorum sensing. *Int. J. Antimicrob. Agents* 42 519–523. 10.1016/j.ijantimicag.2013.07.006 24016798

[B12] Flemming H. C. Wingender J. (2010). The biofilm matrix. *Nat. Rev. Microbiol.* 8 623–633. 10.1038/nrmicro2415 20676145

[B13] Gowrishankar S. Duncun Mosioma N. Karutha Pandian S. (2012). Coral-associated bacteria as a promising antibiofilm agent against methicillin-resistant and -susceptible *Staphylococcus aureus* Biofilms. *Evid Based Complement. Alternat. Med.* 2012:862374 10.1155/2012/862374 PMC3439993 22988476

[B14] Gursoy U. K. Pollanen M. Kononen E. Uitto V. J. (2010). Biofilm formation enhances the oxygen tolerance and invasiveness of *Fusobacterium nucleatum* in an oral mucosa culture model. *J. Periodontol.* 81 1084–1091. 10.1902/jop.2010.090664 20350156

[B15] He Z. Liang J. Tang Z. Ma R. Peng H. Huang Z. (2015). Role of the luxS gene in initial biofilm formation by *Streptococcus mutans*. *J. Mol. Microbiol. Biotechnol.* 25 60–68. 10.1159/000371816 25766758

[B16] He Z. Wang Q. Hu Y. Liang J. Jiang Y. Ma R. (2012). Use of the quorum sensing inhibitor furanone C-30 to interfere with biofilm formation by *Streptococcus mutans* and its luxS mutant strain. *Int. J. Antimicrob. Agents* 40 30–35. 10.1016/j.ijantimicag.2012.03.016 22578766

[B17] Hennequin C. Forestier C. (2009). oxyR, a LysR-type regulator involved in *Klebsiella pneumoniae* mucosal and abiotic colonization. *Infect. Immun.* 77 5449–5457. 10.1128/IAI.00837-09 19786563 PMC2786449

[B18] Hippe B. Zwielehner J. Liszt K. Lassl C. Unger F. Haslberger A. G. (2011). Quantification of butyryl CoA:acetate CoA-transferase genes reveals different butyrate production capacity in individuals according to diet and age. *FEMS Microbiol. Lett.* 316 130–135. 10.1111/j.1574-6968.2010.02197.x 21204931

[B19] Jacqueline C. Caillon J. (2014). Impact of bacterial biofilm on the treatment of prosthetic joint infections. *J. Antimicrob. Chemother.* 69(Suppl. 1) i37–i40. 10.1093/jac/dku254 25135088

[B20] Keskin M. Kononen E. Soderling E. Isik G. Firatli E. Uitto V. J. (2014). Increased proliferation and decreased membrane permeability as defense mechanisms of *Fusobacterium nucleatum* against human neutrophilic peptide-1. *Anaerobe* 30 35–40. 10.1016/j.anaerobe.2014.08.001 25132418

[B21] Kolenbrander P. E. Palmer R. J. Jr. Rickard A. H. Jakubovics N. S. Chalmers N. I. Diaz P. I. (2006). Bacterial interactions and successions during plaque development. *Periodontology* 2000 47–79. 10.1111/j.1600-0757.2006.00187.x 16930306

[B22] Krysciak D. Schmeisser C. Preuss S. Riethausen J. Quitschau M. Grond S. (2011). Involvement of multiple loci in quorum quenching of autoinducer I molecules in the nitrogen-fixing symbiont *Rhizobium* (*Sinorhizobium*) sp. strain NGR234. *Appl. Environ. Microbiol.* 77 5089–5099. 10.1128/AEM.00112-11 21642401 PMC3147472

[B23] Lee H. R. Rhyu I. C. Kim H. D. Jun H. K. Min B. M. Lee S. H. (2011). In-vivo-induced antigenic determinants of *Fusobacterium nucleatum* subsp. nucleatum. *Mol. Oral Microbiol.* 26 164–172. 10.1111/j.2041-1014.2010.00594.x 21375706

[B24] Lee H. S. Gu F. Ching S. M. Lam Y. Chua K. L. (2010). CdpA is a *Burkholderia pseudomallei* cyclic di-GMP phosphodiesterase involved in autoaggregation, flagellum synthesis, motility, biofilm formation, cell invasion, and cytotoxicity. *Infect. Immun.* 78 1832–1840. 10.1128/IAI.00446-09 20194589 PMC2863505

[B25] Lee J. H. Kim Y. G. Ryu S. Y. Cho M. H. Lee J. (2014). Resveratrol oligomers inhibit biofilm formation of *Escherichia coli* O157:H7 and *Pseudomonas aeruginosa*. *J. Nat. Prod.* 77 168–172. 10.1021/np400756g 24456071

[B26] Lee J. H. Kim Y. G. Yong Ryu S. Lee J. (2016). Calcium-chelating alizarin and other anthraquinones inhibit biofilm formation and the hemolytic activity of *Staphylococcus aureus*. *Sci. Rep.* 6:19267 10.1038/srep19267 PMC4725881 26763935

[B27] Lee K. Lee J. H. Ryu S. Y. Cho M. H. Lee J. (2014). Stilbenes reduce *Staphylococcus aureus* hemolysis, biofilm formation, and virulence. *Foodborne Pathog. Dis.* 11 710–717. 10.1089/fpd.2014.1758 25007234

[B28] Lemos J. A. Luzardo Y. Burne R. A. (2007). Physiologic effects of forced down-regulation of dnaK and groEL expression in *Streptococcus mutans*. *J. Bacteriol.* 189 1582–1588. 10.1128/JB.01655-06 17172345 PMC1855735

[B29] Maddocks S. E. Oyston P. C. (2008). Structure and function of the LysR-type transcriptional regulator (LTTR) family proteins. *Microbiology* 154 3609–3623. 10.1099/mic.0.2008/022772-0 19047729

[B30] Miao L. Liu Y. Li Q. Wang Z. Li H. Zhang G. (2010). Screening and sequence analysis of the hemolysin gene of *Fusobacterium necrophorum*. *Anaerobe* 16 402–404. 10.1016/j.anaerobe.2010.04.005 20452448

[B31] Millhouse E. Jose A. Sherry L. Lappin D. F. Patel N. Middleton A. M. (2014). Development of an in vitro periodontal biofilm model for assessing antimicrobial and host modulatory effects of bioactive molecules. *BMC Oral Health* 14:80 10.1186/1472-6831-14-80 PMC4080992 24972711

[B32] Molhoek E. M. Van Dijk A. Veldhuizen E. J. Haagsman H. P. Bikker F. J. (2011). A cathelicidin-2-derived peptide effectively impairs *Staphylococcus epidermidis* biofilms. *Int. J. Antimicrob. Agents* 37 476–479. 10.1016/j.ijantimicag.2010.12.020 21376541

[B33] Nyenje M. E. Green E. Ndip R. N. (2012). Biofilm formation and adherence characteristics of *Listeria ivanovii* strains isolated from ready-to-eat foods in Alice, South Africa. *Sci. World J.* 2012:873909 10.1100/2012/873909 PMC3541635 23365535

[B34] Pei R. Lamas-Samanamud G. R. (2014). Inhibition of biofilm formation by T7 bacteriophages producing quorum-quenching enzymes. *Appl. Environ. Microbiol.* 80 5340–5348. 10.1128/AEM.01434-14 24951790 PMC4136088

[B35] Qin N. Tan X. Jiao Y. Liu L. Zhao W. Yang S. (2014). RNA-Seq-based transcriptome analysis of methicillin-resistant *Staphylococcus aureus* biofilm inhibition by ursolic acid and resveratrol. *Sci. Rep.* 4:5467 10.1038/srep05467 PMC4073122 24970710

[B36] Rezzonico F. Duffy B. (2008). Lack of genomic evidence of AI-2 receptors suggests a non-quorum sensing role for luxS in most bacteria. *BMC Microbiol.* 8:154 10.1186/1471-2180-8-154 PMC2561040 18803868

[B37] Sasaki-Imamura T. Yano A. Yoshida Y. (2010). Production of indole from L-tryptophan and effects of these compounds on biofilm formation by *Fusobacterium nucleatum* ATCC 25586. *Appl. Environ. Microbiol.* 76 4260–4268. 10.1128/AEM.00166-10 20472741 PMC2897440

[B38] Seaton K. Ahn S. J. Sagstetter A. M. Burne R. A. (2011). A transcriptional regulator and ABC transporters link stress tolerance, (p)ppGpp, and genetic competence in *Streptococcus mutans*. *J. Bacteriol.* 193 862–874. 10.1128/JB.01257-10 21148727 PMC3028664

[B39] Sintim H. O. Gursoy U. K. (2016). Biofilms as “connectors” for oral and systems medicine: a new opportunity for biomarkers, molecular targets, and bacterial eradication. *OMICS* 20 3–11. 10.1089/omi.2015.0146 26583256 PMC4739346

[B40] Skar C. K. Kruger P. G. Bakken V. (2003). Characterisation and subcellular localisation of the GroEL-like and DnaK-like proteins isolated from *Fusobacterium nucleatum* ATCC 10953. *Anaerobe* 9 305–312. 10.1016/j.anaerobe.2003.08.004 16887717

[B41] Srinandan C. S. Elango M. Gnanadhas D. P. Chakravortty D. (2015). Infiltration of matrix-non-producers weakens the salmonella biofilm and impairs its antimicrobial tolerance and pathogenicity. *Front. Microbiol.* 6:1468 10.3389/fmicb.2015.01468 PMC4688346 26779121

[B42] Tian Y. He X. Torralba M. Yooseph S. Nelson K. E. Lux R. (2010). Using DGGE profiling to develop a novel culture medium suitable for oral microbial communities. *Mol. Oral Microbiol.* 25 357–367. 10.1111/j.2041-1014.2010.00585.x 20883224 PMC2951289

[B43] Trappetti C. Potter A. J. Paton A. W. Oggioni M. R. Paton J. C. (2011). LuxS mediates iron-dependent biofilm formation, competence, and fratricide in *Streptococcus pneumoniae*. *Infect. Immun.* 79 4550–4558. 10.1128/IAI.05644-11 21875962 PMC3257940

[B44] Trentin D. S. Silva D. B. Frasson A. P. Rzhepishevska O. Da Silva M. V. Pulcini Ede L. (2015). Natural Green coating inhibits adhesion of clinically important bacteria. *Sci. Rep.* 5:8287 10.1038/srep08287 PMC4319173 25655943

[B45] van den Bogaard A. E. Stobberingh E. E. (2000). Epidemiology of resistance to antibiotics. Links between animals and humans. *Int. J. Antimicrob. Agents* 14 327–335. 10.1016/S0924-8579(00)00145-X 10794955

[B46] Vanderlinde E. M. Harrison J. J. Muszynski A. Carlson R. W. Turner R. J. Yost C. K. (2010). Identification of a novel ABC transporter required for desiccation tolerance, and biofilm formation in *Rhizobium leguminosarum* bv. viciae 3841. *FEMS Microbiol. Ecol.* 71 327–340. 10.1111/j.1574-6941.2009.00824.x 20030718 PMC2868943

[B47] Yu J. Ge J. Heuveling J. Schneider E. Yang M. (2015). Structural basis for substrate specificity of an amino acid ABC transporter. *Proc. Natl. Acad. Sci. U.S.A.* 112 5243–5248. 10.1073/pnas.1415037112 25848002 PMC4413293

[B48] Yuan L. Hillman J. D. Progulske-Fox A. (2005). Microarray analysis of quorum-sensing-regulated genes in *Porphyromonas gingivalis*. *Infect. Immun.* 73 4146–4154. 10.1128/iai.73.7.4146-4154.2005 15972504 PMC1168601

[B49] Zhang P. Chomel B. B. Schau M. K. Goo J. S. Droz S. Kelminson K. L. (2004). A family of variably expressed outer-membrane proteins (Vomp) mediates adhesion and autoaggregation in *Bartonella quintana*. *Proc. Natl. Acad. Sci. U.S.A.* 101 13630–13635. 10.1073/pnas.0405284101 15347808 PMC518805

