# Supplementary figures and images for: Anti-biofilm Activities from Resveratrol against Fusobacterium nucleatum
Source: Front Microbiol. 2016 Jul 5;7:1065. doi: 10.3389/fmicb.2016.01065 (PMC4932316; doi:10.3389/fmicb.2016.01065)

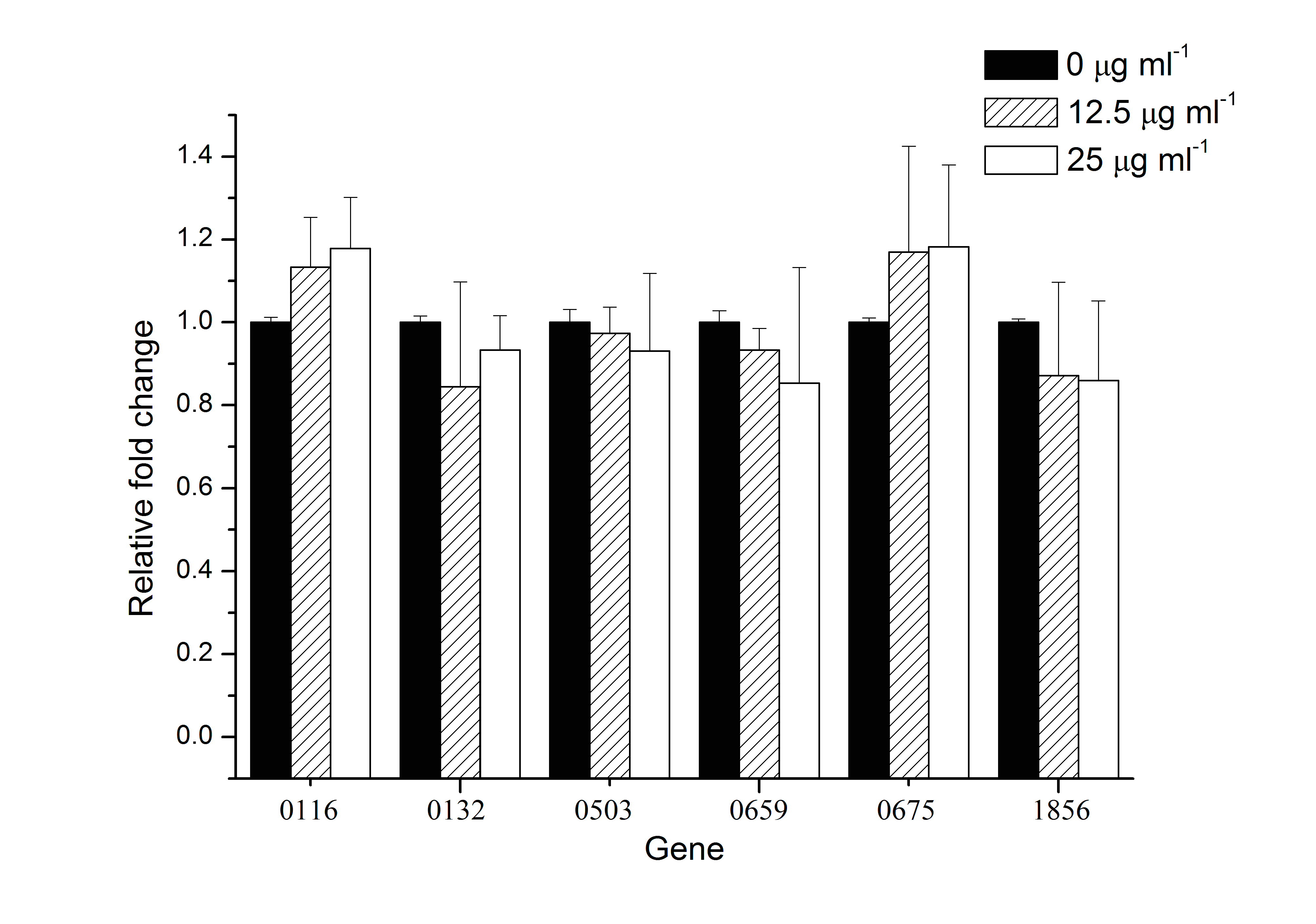

Supplement: FIGURE S1 — Gene expression of F. nucleatum strain planktonic cultures. The results represent the means and SDs of three independent experiments performed in triplicate. No significant differences (P > 0.05) in gene expression between different concentrations. [file Image_1.TIF]
